# Acute pulmonary edema secondary to magnesium sulfate tocolysis in twin pregnancy: a case report

**DOI:** 10.1515/crpm-2025-0029

**Published:** 2026-06-01

**Authors:** Xiaoling Liang, Jiaxin Kang, Bowen Zheng, Yicong Liao, Shiqing Wang, Guoliang Yan, Junxiang Jia

**Affiliations:** Department of Anesthesiology, Department of Obstetrics and Gynecology, Women and Children’s Hospital, School of Medicine, Xiamen University, Xiamen, Fujian, China; School of Medicine, Xiamen University, Xiamen, Fujian, China

**Keywords:** pulmonary edema, magnesium sulfate, tocolysis, twin pregnancy, maternal health, perinatal complications

## Abstract

**Objectives:**

Acute pulmonary edema during pregnancy is a rare but serious complication, particularly in association with magnesium sulfate therapy for tocolysis. This case report describes a 27-year-old woman with a twin pregnancy who developed acute pulmonary edema after 3 days of magnesium sulfate treatment.

**Case presentation:**

The patient presented with chest tightness, dyspnea, and respiratory distress. Immediate clinical intervention included discontinuation of magnesium sulfate and administration of furosemide to reduce cardiac preload. She was managed with diuretics, sedatives, and inotropic agents. Both infants were delivered successfully, and the patient recovered after supportive care in the intensive care unit.

**Conclusions:**

This case highlights the potential risks of fluid retention and the importance of monitoring maternal fluid status during tocolysis. It also emphasizes the need for vigilance in managing twin pregnancies with magnesium sulfate therapy to prevent serious complications such as pulmonary edema. Clinicians should be aware of this rare but potentially life-threatening complication and ensure that fluid balance is carefully monitored in such cases.

## Introduction

Peripartum pulmonary edema is a rare but serious condition with a reported incidence of 0.08 % [1]. Common causes include hypertensive disorders of pregnancy, cardiac disease, and fluid overload, while rare causes comprise amniotic fluid embolism, thyroid storm, and neurogenic pulmonary edema. Preeclampsia and peripartum cardiomyopathy are the most common causes of acute respiratory failure in pregnancy [2], [3], [4]. Differential diagnosis should include viral myocarditis and cardiomyopathy, particularly peripartum cardiomyopathy [5], [6], [7]. During pregnancy, maternal cardiac output increases, heart rate accelerates, colloid osmotic pressure decreases, and total body fluid volume naturally increases. These physiological changes collectively increase the risk of pulmonary edema [8]. Currently, no specific treatment exists for perinatal pulmonary edema. Management relies on early recognition, prompt diagnosis, timely intervention, and prevention of disease progression.

In this report, we present the case of a 27-year-old woman with a twin pregnancy and no significant medical history who developed acute pulmonary edema 3 days after receiving magnesium sulfate therapy for tocolysis.

## Case presentation

A 27-year-old pregnant woman with no significant medical history was admitted to our hospital at 33 weeks and 4 days of a twin gestation due to threatened preterm labor. During pregnancy, the patient’s blood pressure was normal, with no dizziness, headache, chest tightness, chest pain, palpitations, fever, cough, or dyspnea. After admission, intravenous magnesium sulfate (1 g/h, 15 g/day) was administered for fetal neuroprotection, which is within the typical safe clinical range (25–30 g/day) [9]. Betamethasone was given to promote fetal lung maturity. Post-admission echocardiography showed mild mitral and tricuspid regurgitation, with a normal ejection fraction and wall motion. A routine electrocardiogram was normal. Liver and kidney function, myocardial enzymes, and troponin levels were also normal. (Routine examination results were normal; please refer to the attachment.).

After 3 days of treatment, the patient suddenly developed chest tightness and dyspnea. Her vital signs were as follows: 10 L/min O_2_ via a simple mask, SpO_2_ 80 %, heart rate 120 bpm, respiratory rate 26 breaths/min, and blood pressure 149/96 mmHg. Auscultation revealed moist rales in both lungs, with the left lung more prominently affected. Based on the patient’s clinical presentation, acute pulmonary edema was suspected.

Magnesium sulfate therapy was discontinued immediately. The patient received 20 mg of intravenous furosemide to reduce cardiac preload; the head of the bed was elevated to a semi-recumbent position; a complete coagulation profile, N-terminal pro-B-type natriuretic peptide (BNP), cardiac enzymes, and troponin tests were ordered; emergency electrolytes were administered; and she was prepared for an emergency cesarean section.

Upon entering the operating room, she remained in a semi-recumbent position, exhibiting dyspnea, altered consciousness, and cyanosis of the lips and face, and no rash. At that time, her blood pressure was 193/110 mmHg, heart rate was 155 bpm, respiratory rate was 30 breaths/min, and SpO_2_ was 45 %. General anesthesia was induced with 120 mg propofol, 120 μg remifentanil, and 10 mg cisatracurium.

Tracheal intubation was performed using a video laryngoscope, revealing a large amount of foamy sputum in the oropharynx. Pink frothy sputum was also observed in the endotracheal tube. Auscultation revealed markedly diminished breath sounds in the left lung and moist rales in the right lung. Following induction, SpO_2_ gradually increased to 98 %, blood pressure stabilized at 113/61 mmHg, and heart rate decreased from 150 to 125 bpm. Based on the clinical presentation, the patient was diagnosed with acute pulmonary edema.

Two healthy babies were successfully delivered, with Apgar scores of 6-8-10 and 8-9-10 at 1, 5, and 10 min after birth, respectively. Anesthesia was deepened with 3 mg of midazolam and 30 μg of sufentanil and maintained using continuous infusions of propofol and remifentanil. The patient was administered 0.2 mg of cedilanid intravenously to improve cardiac contractility and 2 mg of morphine for its antispasmodic and analgesic effects.

An arteriovenous catheter was inserted to monitor the arterial and central venous pressure. Arterial blood gas analysis (on 100 % FiO_2_) revealed a pH of 7.25, PaCO_2_ of 58.7 mmHg, and PaO_2_ of 169 mmHg (Table 1). In addition, the patient was administered 80 mg of intravenous methylprednisolone to reduce capillary permeability and 20 mg of furosemide to promote diuresis. These interventions led to significant clinical improvement.

**Table 1:** Blood gas analysis and vital indicators over time.

Test indicators	Time					
	12.24 preoperative	12.24 intraoperative	12.24 Urgent postoperative examination	12.24 postoperative (after extubation)	12.25 am	12.29
pH	/	7.25	7.31	7.4	/	/
PaO_2_, mmHg	/	169	155	145	/	/
PaCO_2_, mmHg	/	58.7	55.4	37.7	/	/
FiO_2_, %	/	100	75	41	/	/
PaO_2_/FiO_2_	/	169	206	353	/	/
BNP, pg/mL	748	/	816	588	311	28
Troponin I, ng/L	14.545	/	129.56	245.708	57.568	4.759

BNP, b-type natriuretic peptide; FiO_2_, fraction of inspired oxygen.

By the end of the surgery, the total blood loss was 300 mL. During the procedure, the patient received 200 mL of sodium, potassium magnesium, calcium, and glucose solution intravenously, as well as 50 mL of hydroxyethyl starch. Her total urine output was 700 mL.

**Figure 1:**
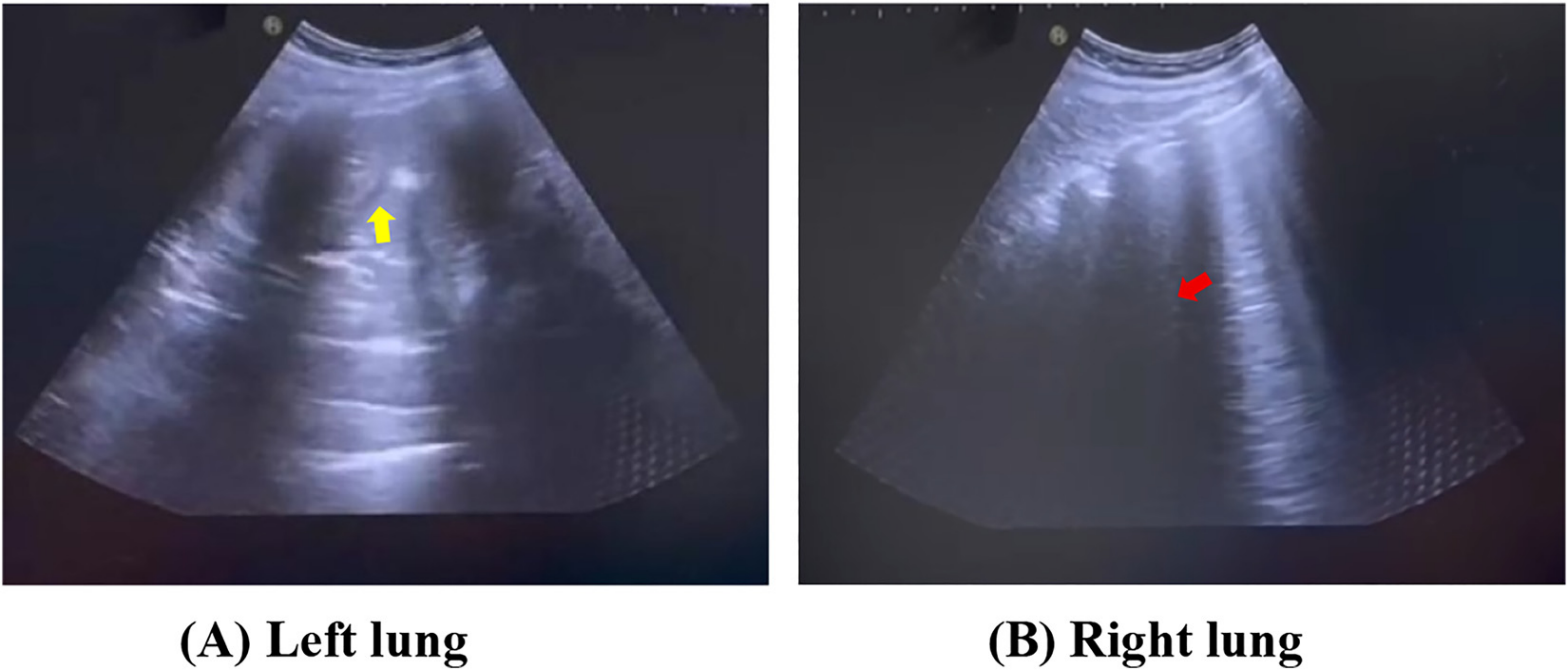
Bedside lung ultrasound performed immediately after surgery. (A) Left lung consolidation (yellow arrow). (B) Numerous B-lines in the right lung (red arrow).

Results from intraoperative and immediate postoperative urgent testing revealed markedly elevated levels of high-sensitivity troponin and BNP (Table 1). Immediately after surgery, while the patient was still in the operating room, bedside lung ultrasonography showed numerous B-lines in the right lung and consolidation-like changes in the left lung, which were consistent with acute pulmonary edema (Figure 1). Bedside ultrasound also showed an ejection fraction of 63 %, mild mitral and tricuspid regurgitation, and normal left ventricular systolic function (Routine examination results were normal; please refer to the attachment.).

**Figure 2:**
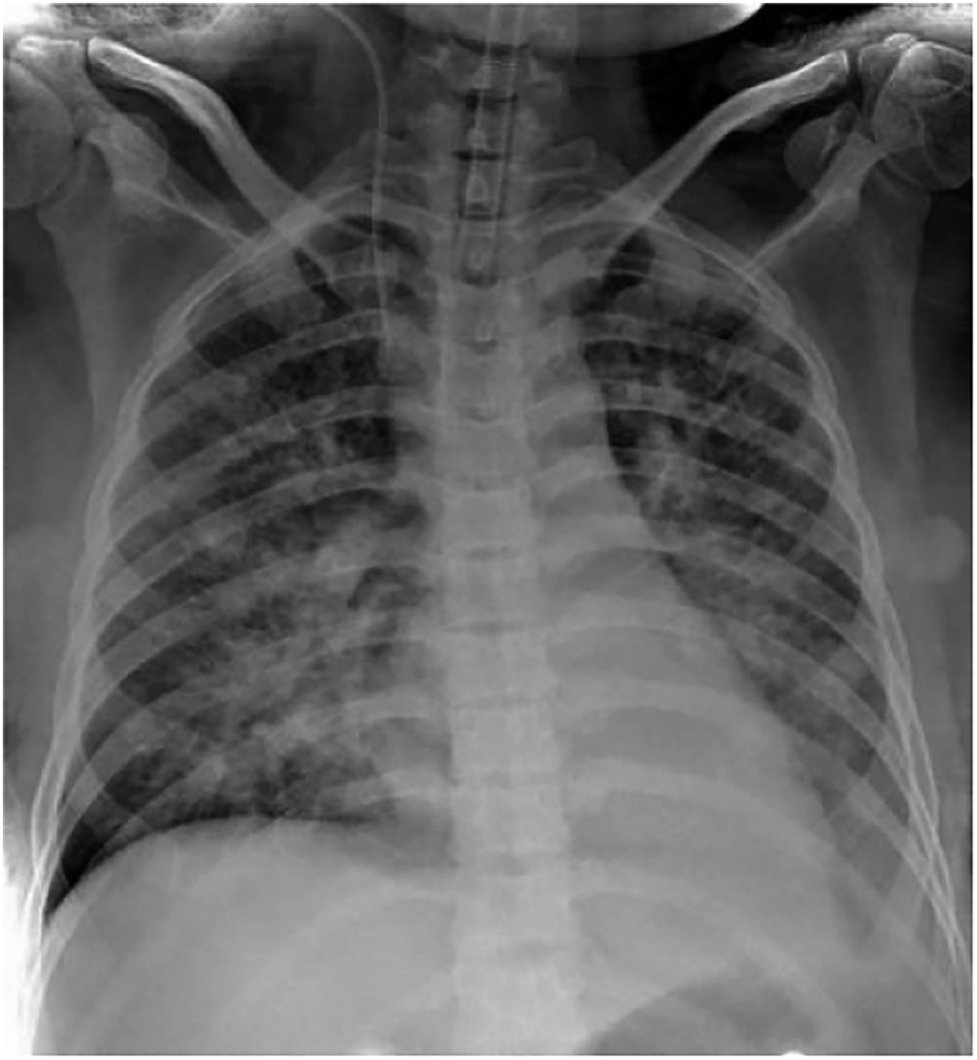
Chest X-ray chest X-ray. Bilateral lung field translucency is unevenly decreased, bilateral lung texture is increased and thickened, and multiple patches and speckled hyperdense shadows with unclear borders can be seen. Multiple foci of disease are present in both lungs, and the possibility of pulmonary edema is considered in conjunction with the clinical picture.

Bedside chest radiography revealed increased thickening of bilateral lung markings, heterogeneous reduction in bilateral lung field brightness, and multiple patchy and speckled areas of increased density with poorly defined margins. The right hilar shadow appeared enlarged and thickened, while the left hilar shadow and mediastinum were indistinct. A mildly enlarged cardiac silhouette was also noted (Figure 2).

The ancillary findings further supported the diagnosis of acute pulmonary edema, and 10 μg of sufentanil was administered intravenously for pain relief. |After 30 min, blood gas analysis (on 75 % FiO_2_) revealed an increased oxygenation index and improved gas exchange capacity (Table 1), suggesting resolution of the alveolar interstitial edema. Additionally, 20 mg of furosemide was administered intravenously to promote diuresis.

One hour after surgery, the patient’s vital signs were stable and auscultation revealed a significant improvement in lung sounds. She was subsequently transferred to the intensive care unit (ICU) with an endotracheal tube *in situ* for continued observation and treatment. In the ICU, mechanical ventilation was maintained, and sedation was provided with midazolam and propofol. Sodium nitroprusside was administered for blood pressure control.

Propofol infusion was discontinued 3 h postoperatively, and the patient regained consciousness. The endotracheal tube was successfully removed 6 h postoperatively; the patient exhibited no symptoms such as dyspnea, palpitations, or chest tightness. However, rales were still present on lung auscultation. Furosemide was continued for diuresis, and albumin was administered for volume support. Sodium nitroprusside was maintained to control blood pressure.

On postoperative day 2, oral nifedipine controlled-release tablets (30 mg once daily) were initiated for hypertension management. Following this, her high-sensitivity troponin and BNP levels gradually returned to normal (Table 1), and lung function normalized. She achieved a full recovery and was discharged on postoperative day 7.

## Discussion

This report outlines the case of a 27-year-old woman with a twin pregnancy and no significant medical history. The patient received intravenous magnesium sulfate (1 g/h, 15 g/d) for fetal neuroprotection [9]; although the typical clinical dose of magnesium sulfate is 25–30 g/day and the patient’s dosage was within this safe range, she developed sudden-onset, life-threatening pulmonary edema.

Pulmonary edema during pregnancy typically results from multiple factors, and distinguishing its triggers and types is crucial for appropriate treatment. The most common reported cause of perinatal pulmonary edema is hypertensive disorders of pregnancy (preeclampsia) [3]. However, in the current patient, blood pressure elevation occurred after the onset of pulmonary edema (likely a reactive change due to hypoxia and stress), and there were no signs of proteinuria or hepatic/renal impairment, which does not align with the features of pulmonary edema secondary to a hypertensive emergency.

Regarding cardiogenic factors, conditions such as peripartum cardiomyopathy (PPCM), viral myocarditis, or acute coronary syndrome can lead to heart failure and pulmonary edema. PPCM is a rare, life-threatening condition characterized by rapidly developing heart failure in the final month of pregnancy or within 5 months postpartum, primarily featuring reduced left ventricular systolic function [5], 6]. The current patient was a young woman with no cardiovascular history, and pre- and post-operative echocardiography showed normal left ventricular ejection fraction and no wall motion abnormalities. Her preoperative troponin and electrocardiogram were normal, and cardiopulmonary function recovered rapidly within 6 h, which ruled out these cardiogenic causes.

With regards to non-cardiogenic factors, drug-induced hypersensitivity reactions such as DRESS syndrome can manifest with pulmonary involvement including interstitial infiltrates, pneumonia, pleural effusion, and acute respiratory distress syndrome (ARDS). Typical clinical features include fever, rash, lymphadenopathy, peripheral eosinophilia, and multi-organ involvement, typically with a latency of 2–8 weeks after drug initiation [10]. Although this patient received magnesium sulfate, symptoms appeared just 3 days after administration, which is inconsistent with the typical latency. Moreover, she had no fever, rash, or eosinophilia, thus excluding drug-induced hypersensitivity-related ARDS.

During her twin pregnancy, the patient’s blood volume naturally increased, and magnesium sulfate administration may have contributed to sodium and water retention. Further review of her medical history revealed that she had reported excessive thirst on admission, with daily water intake reaching 2000–3,000 mL. We hypothesize that the combination of twin pregnancy, magnesium sulfate used for tocolysis, and high fluid intake led to a sharp increase in intravascular volume, ultimately triggering acute pulmonary edema.

This case highlights the importance of strict fluid management in patients receiving medications that may disrupt water-sodium balance, even when administered at safe doses. In the context of multiple gestations, clinicians should strengthen prenatal monitoring and patient education. Daily fluid intake and output should be carefully monitored, documented, and interpreted alongside clinical signs and laboratory findings to comprehensively evaluate maternal fluid status and prevent complications such as pulmonary edema.

Additionally, for pregnant patients presenting with early symptoms, such as dyspnea or chest tightness, we recommend the use of bedside cardiac and lung ultrasound as an early diagnostic tool. Prompt identification and intervention can be lifesaving in such cases.

## Conclusions

We report a case of perinatal acute pulmonary edema triggered by inadequate prenatal fluid monitoring and management in a woman with a twin pregnancy who was treated solely with magnesium sulfate. Particular attention must be paid to fluid management and monitoring of spontaneous water intake when administering magnesium sulfate in the context of multiple gestation to prevent the development of acute pulmonary edema.

## Supplementary Material

Supplementary Material
