# Supplementary material for: Acute pulmonary edema secondary to magnesium sulfate tocolysis in twin pregnancy: a case report
Source: Case Rep Perinat Med. 2026 Jun 1;15(1):20250029. doi: 10.1515/crpm-2025-0029 (PMC13225067; doi:10.1515/crpm-2025-0029)
Supplement: Supplementary file 1 — Supplementary Material [file j_crpm-2025-0029_suppl_001.doc]

**Attachment**

**Acute pulmonary edema secondary to magnesium sulfate tocolysis in twin pregnancy: a case report**

**Content**

[1.Electrocardiogram on admission: 2](#_Toc4734)

[2.Transthoracic echocardiography on admission: 3](#_Toc5713)

[3.Bedside transthoracic echocardiography post-surgery: 4](#_Toc6742)

**1. Electrocardiogram on admission:**


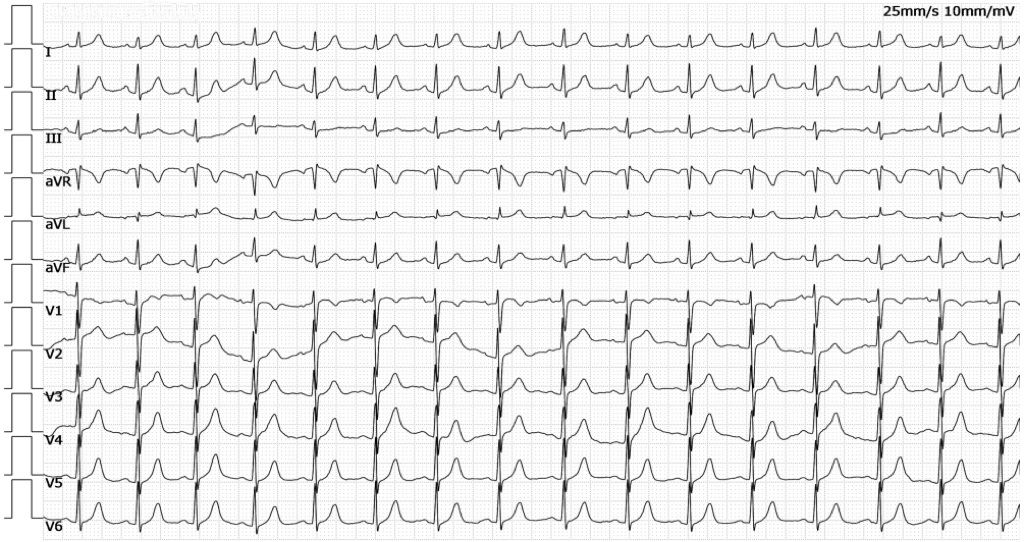
**Description:** P waves are upright in leads I, II, and aVF, and inverted in lead aVR. The R-R intervals are regular. The QRS complexes are supraventricular in morphology. No significant ST-T wave abnormalities are observed.

**Impression:** 1. Sinus rhythm. 2. Normal electrocardiogram.

**2. Transthoracic echocardiography on admission:**


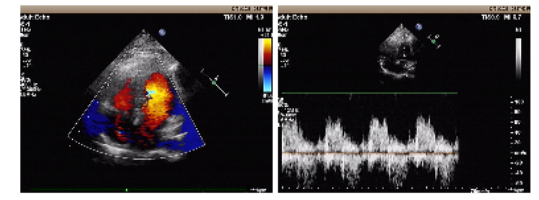


**Measurements:**

| Parameter | Value | Reference range |  | Parameter | Value | Reference range |
| --- | --- | --- | --- | --- | --- | --- |
| A0 (cm) | 2.6 | <3.8 cm |  | LA (cm) | 2.9 | <3.7 cm |
| RV (cm) | 2.9 | <4.0 cm |  | lVS (cm) | 0.89 | <1.1 cm |
| LVDd (cm) | 4.4 | <5.5 cm |  | LVPWD (cm) | 0.90 | <1.2 cm |
| LVDs (cm) | 3.0 | <3.6 cm |  | PA (cm) | 2.6 | <2.8 cm |
| EF (%) | 60 | 50-90% |  |  |  |  |

**Description:** The connections between the cardiac chambers and great vessels are normal. The sizes of all cardiac chambers and the internal diameters of the great vessels are within normal limits. The atrial and ventricular septa appear continuous, with no significant echo dropout observed. All cardiac valves exhibit normal opening and closing motions. Color Doppler Flow Imaging reveals trace regurgitant signals at the mitral and tricuspid valves; no significant abnormal flow signals are detected in other valvular areas. The E/A ratio is greater than 1 (E/A >1).

**Impression:** Mild mitral and tricuspid regurgitation.

**3. Bedside transthoracic echocardiography post-surgery:**

**
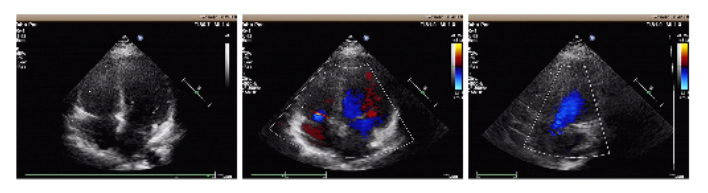
**

**Measurements:**

| Parameter | Value | Reference range |  | Parameter | Value | Reference range |
| --- | --- | --- | --- | --- | --- | --- |
| A0 (cm) | 2.7 | <3.8 cm |  | LA (cm) | 3.0 | <3.7 cm |
| RV (cm) | 2.9 | <4.0 cm |  | lVS (cm) | 0.9 | <1.1 cm |
| LVDd (cm) | 4.3 | <5.5 cm |  | LVPWD (cm) | 0.90 | <1.2 cm |
| LVDs (cm) | 2.8 | <3.6 cm |  | PA (cm) | 2.4 | <2.8 cm |
| EF (%) | 63 | 50-90% |  |  |  |  |

**Description:** The anatomical connections between the cardiac chambers and great vessels are normal. The dimensions of all cardiac chambers and the internal diameters of the great vessels are within normal limits. The atrial and ventricular septa are continuous, with no evidence of septal defects or significant echo dropout. Valvular leaflets exhibit normal opening and closing excursions. Color Doppler Flow Imaging demonstrates trace regurgitant signals at the mitral and tricuspid valves, with a tricuspid regurgitation pressure gradient of 18 mmHg. No significant abnormal flow signals are observed in other valvular areas. The E/A ratio is greater than 1 (E/A >1).

**Impression:** Mild mitral and tricuspid regurgitation. Normal left ventricular systolic function.
